# The Effect of Bariatric Surgery Prior to Lower-Extremity Total Joint Arthroplasty: A Systematic Review

**DOI:** 10.1007/s11420-019-09674-2

**Published:** 2019-04-09

**Authors:** Alex Gu, Jordan S. Cohen, Michael-Alexander Malahias, Danny Lee, Peter K. Sculco, Alexander S. McLawhorn

**Affiliations:** 1 George Washington University School of Medicine and Health Sciences, 2300 Eye St. NW, Washington, DC 20037 USA; 2 Stavros Niarchos Foundation Complex Joint Reconstruction Center, Hospital for Special Surgery, 535 E. 70th St, New York, NY 10021 USA; 3 Adult Reconstruction and Joint Replacement, Hospital for Special Surgery, 535 E. 70th St, New York, NY 10021 USA

**Keywords:** bariatric surgery, obesity, total knee arthroplasty, post-operative complications

## Abstract

**Background:**

Obesity is an independent risk factor for osteoarthritis and has been associated with increased rate of complications following lower-extremity total joint arthroplasty (TJA). Bariatric surgery (BS) is a surgical option for weight loss and for reducing obesity-related comorbidities in morbidly obese patients.

**Purpose/Questions:**

The goal of this systematic review was to answer the following questions: (1) Does BS prior to TJA correlate with lower post-operative complication rates in morbidly obese patients undergoing TJA? (2) Does BS have an impact on revision rates following TJA?

**Methods:**

Using the Preferred Reporting Items for Systematic Reviews and Meta-Analyses (PRISMA) statement and checklist, a systematic review of medical databases (PubMed/ MEDLINE, Cochrane Library, Web of Science, and Clinicaltrials.gov) was undertaken for articles published in English from January 1990 to September 2018. Inclusion criteria were studies that included at least ten patients who underwent BS prior to TJA, collected data on complications or other outcomes, and followed patients for at least 90 days after TJA. A descriptive and critical analysis of the results was performed.

**Results:**

From 799 studies, 13 met inclusion criteria. A total of 11,770 patients who had undergone bariatric surgery prior to TJA were analyzed. The quality of the evidence ranged between moderate and high. There was no consensus on the effect of previous BS on early- to short-term outcomes reported after TJA.

**Conclusion:**

The literature remains conflicted on the impact of BS prior to TJA on early, short-term, and long-term complications after TJA. Additional well-matched, observational studies may further our understanding of the impact of BS prior to TJA on outcomes. In particular the effect of various types of BS prior to TJA on outcomes has yet to be elucidated. Ideally, prospective studies with higher level of evidence will be more definitive on the effects of BS prior to TJA.

**Prospero Registration Number:** CRD42016043025.

**Electronic supplementary material:**

The online version of this article (10.1007/s11420-019-09674-2) contains supplementary material, which is available to authorized users.

## Introduction

Obesity, in particular morbid obesity, has been increasing in the USA at an alarming rate [39]. Obesity is defined as a body mass index (BMI) of greater than 30 kg/m^2^, while morbid obesity is defined as a BMI of greater than 40 kg/m^2^; obesity is associated with numerous comorbidities, including type 2 diabetes, cardiovascular diseases, and chronic back pain [6, 7].

In addition, obesity has been widely accepted as an independent risk factor for osteoarthritis of the hip and knee [2–4, 9, 13, 30, 34]. As a final treatment, total hip arthroplasty (THA) and total knee arthroplasty (TKA) remain the gold standard treatments for osteoarthritis of the hip and knee [12]. The rates of THA and TKA performed has increased along with the increase in rates of obesity [12]. A recent study found that 56.5% of patients who undergo TKA as a result of severe osteoarthritis are obese [27]. Obesity has been associated with worse clinical outcomes after total joint arthroplasty (TJA), including poor wound healing, increased infection rate, and post-operative stiffness [11, 44].

Weight loss strategies used in the treatment of obesity include lifestyle modification and pharmacotherapy [23, 25]. However, for morbidly obese patients having inadequate weight loss from medical weight management, bariatric surgery (BS) is an option. The strongest indication for BS is a BMI greater than 40 kg/m^2^, or BMI greater than 35 kg/m^2^ with an obesity-related comorbidity, such as hypertension or diabetes [25]. Compared to patients attempting non-surgical weight loss, people who undergo BS are more likely to experience greater, more sustainable weight loss at long-term follow-up [18, 23]. Furthermore, BS has been shown to reduce obesity-related comorbidities [5, 21, 35]. Recently, more clinical studies have been published investigating the associations between BS and lower-extremity TJA outcomes. The goal of this study was to review the literature regarding BS prior to TJA. Specifically, we aimed to answer the following questions: (1) Is BS prior to TJA correlated with lower post-operative complication rates in morbidly obese patients undergoing TJA? (2) Does BS have an impact on revision rate following TJA?

## Methods

This review was registered with PROSPERO (no. CRD42016043025). Using the Preferred Reporting Items for Systematic Reviews and Meta-Analyses (PRISMA) statement and checklist [26], a thorough search of electronic databases (MEDLINE/PubMed, Cochrane Library, Web of Science, and ClinicalTrials.gov) was undertaken for articles published from January 1990 to September 2018. The keywords used in the initial screening were split into two groups. The first group of search terms was *bariatric surgery*, *gastric bypass*, *gastric band*, *lap band*, *gastric sleeve*, *duodenal switch*, *Roux-en-Y*, and *surgical weight loss*. The second group of search terms was *total knee arthroplasty*, *knee replacement*, *total hip arthroplasty*, *hip replacement*, and *total joint arthroplasty*. Each database was queried using combinations of these search terms, with each search including one keyword from each group. Two of the authors (A.G. and J.S.C.) independently performed the search and uploaded their search results into a spreadsheet tool. Once completed, they reconciled their findings. See Appendix 1 for more on the search strategy.

The inclusion criteria were (1) studies describing human subjects of any age and gender, (2) studies including a population of at least ten patients who underwent BS (gastric bypass, gastric band, lap band, gastric sleeve, duodenal switch, Roux-en-Y) prior to TJA, (3) studies presenting data concerning complication rates and/or other outcome measures for patients who underwent BS prior to TJA, and (4) studies following patients for a minimum of 90 days after TJA. The exclusion criteria were (1) review articles; (2) case reports; (3) studies examining exclusively non-surgical weight loss strategies; (4) studies stratifying patients based on peri-operative management (anesthesia protocol, limitation of blood loss, surgical technique, prosthesis type, etc.), in which allocation of patients who previously underwent BS is not specified; (5) studies in which subjects did not undergo TJA; and (6) non-English language publications. For articles that met the inclusion criteria, the reference lists were screened for additional studies not captured using the initial search terms.

Two reviewers (A.G. and J.S.C.) extracted data from the articles eligible for study inclusion. During initial data review, the following information was collected for each study: title; author; study design; primary study outcomes; study cohorts; mean age; mean BMI; sex; 30-day complications; 90-day complications; 1-,2-, 5-, or 10-year complications; mortality; outcome rate; revision rate; and hospital length of stay (LOS). Two reviewers independently evaluated the level of evidence using the Oxford Centre for Evidence-based Medicine (CEBM) guidelines [45], a widely used tool for study appreciation, allowing for comparison of studies based on their design. Only studies with a CEBM rating of 4 or better were considered. The methodological quality of the reviewed papers was assessed using the revised version of the Methodological Index for Non-Randomized Studies (MINORS) criteria, which allow for the calculation of a score that corresponds to the soundness of the methodology in comparative and non-comparative surgical studies [38]. The maximum MINORS score is 16 for non-comparative studies and 24 for comparative studies. The score for each study was converted to a percentage of the maximum possible score for that study design and evaluated qualitatively in accordance with Yeung et al.’s framework for methodological quality: under 25% (very low quality), 25 to 49% (low quality), 50 to 74% (moderate quality), above 75% (high quality) [46].

## Results

**Fig. 1:**
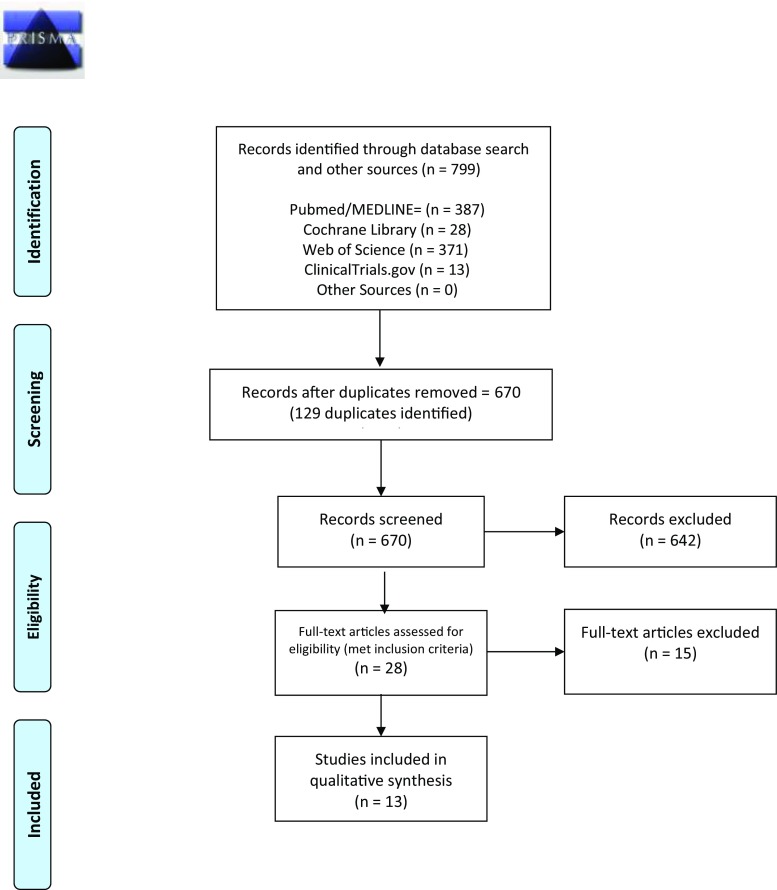
Systematic review flow diagram.

Overall, the database search led to the identification of 799 total studies. In total, 129 duplicates were identified and removed, yielding 670 unique results. A title and abstract screening using the described inclusion and exclusion criteria was performed in duplicate to evaluate all remaining studies. Papers remaining after the title and abstract review were subject to a full-text review. After the full-text review, 13 papers were found to satisfy all inclusion and exclusion criteria (Fig. 1). Table 1 provides an overview of the design, methodological quality, size, and outcomes reported in each included study.

**Table 1:** Overview of studies included in systematic review

Authors	Year	Study time course	CEBM rating	Revised MINORS Score (quality rating) 16 possible points for non-comparative, 24 possible points if comparative	Overall number of patients undergoing TJA	Patients undergoing bariatric surgery before TJA		Notes
						Number	Percent of overall patients	
Parvizi et al. [31]	2000	Retrospective	4	8/16 (moderate quality)	7	7	100%	
Kulkarni et al. [15]	2010	Retrospective	3b	20/24 (high quality)	68	31	45.6%	Some complications data pooled with THA
Severson et al. [37]	2012	Retrospective	3b	16/24 (moderate quality)	125	86	20%	
Werner et al. [43]	2014	Retrospective	3b	19/24 (high quality)	78,036	219	0.3%	
Inacio et al. [17]	2014	Retrospective	3b	19/24 (high quality)	8550 TKA, 2653 THA	134 TKA, 37 THA	0.7%	Complications data pooled with THA
Martin et al. [22]	2015	Retrospective	3b	19/24 (high quality)	364	91	25%	
Nickel et al. [29]	2016	Retrospective	3b	21/24 (high quality)	39,014	5918	15.2%	
Nearing et al. [28]	2016	Retrospective	3b	17/24 (moderate quality)	102	36	35.3%	Data pooled with THA
McLawhorn et al. [24]	2017	Retrospective	3b	19/24 (high quality)	5272	2636	50%	
Schwarzkopf et al. [36]	2017	Retrospective	3b	19/24 (high quality)	1017	1017	100%	
Lee et al. [19]	2018	Retrospective	3b	19/24 (high quality)	86,609	70	0.1%	
Liu et al. [20]	2018	Retrospective	3b	19/24 (high quality)	343,710	1478	25%	Data pooled with THA
Watts et al. [42]	2018	Retrospective	3b	19/24 (high quality)	141	47	33.3%	

*THA* total hip arthroplasty, *TJA* total joint arthroplasty, *TKA* total knee arthroplasty, *CEBM* Centre for Evidence-Based Medicine, *MINORS* Methodological Index for Non-Randomized Studies

In total, 11,770 patients who had BS before TJA were reported. Outcomes studied included early (in-hospital and 30-day) complications, 90-day complications, 1- or 2-year complications, mortality rates, revision rates, and LOS. Of the 13 studies included in this review, 5 (38%) analyzed both THA and TKA after BS [15, 17, 24, 28, 31], while 7 (54%) focused exclusively on TKA [19, 22, 29, 30, 36, 37, 43]. One paper (8%) focused solely on THA [42]. All studies were retrospective. Among the 13 studies, 12 (92.3%) were CEBM level 3b and one study (7.7%) was CEBM level 4 (Table 1). The average MINORS score was 17.6 (73.3%; range 8 to 21). Among the selected studies, three (23.1%) denote reduction in BMI between BS and TJA [22, 31, 42], while 10 (76.9%) did not mention changes in BMI [15, 17, 19, 20, 24, 28, 29, 36, 37, 43].

### Complications

All studies reported post-operative complications, most commonly at less than 30 days [15, 20, 24, 28, 29] or 90 days [15, 17, 20, 24, 29, 36, 37, 43]. Cutoffs of 1 year [17, 19, 20], 2 years [19, 29, 42], or 10 years [42] were reported in five studies (38.5%), while two studies (15.4%) did not provide a specific time frame for when complications occurred [22, 31].

### Peri-Operative and Early (Less than 30 Days) Complications

In the only study that evaluated in-hospital complications [24], the in-hospital complication rate for patients who underwent BS before TKA was lower (2.7%) than the rate in a matched morbidly obese control population (3.9%; *p* = 0.021). The odds ratio for in-hospital complications for patients who underwent BS compared to morbidly obese controls was 0.69 (95% CI 0.51–0.95; *p* = 0.021). Likewise, for THA patients, the odds of in-hospital complications was significantly reduced for patients who had undergone prior BS (OR = 0.25, 95% CI 0.13–0.50) [24].

In total, four studies (30.7%) [15, 20, 28, 29] compared 30-day complications in patients who underwent THA and TKA before BS to those who underwent BS before TJA. In three studies, there was no significant difference between the BS and non-BS groups [15, 20, 28] (Table 2). Nickel et al. compared 30-day complications in patients who underwent BS before TKA to a group of normal-weight individuals and a group of morbidly obese individuals, finding that most complications occurred with the highest frequency in the BS-before-TKA group [29]. Nickel et al. reported significantly higher 30-day complications in patients who had BS before TKA when compared to normal-weight individuals undergoing TKA (*p* ≤ 0.001) [29]. For the same complications, the BS-before-TKA population also had higher rates than morbidly obese controls (*p* ≤ 0.001). Of note, Nickel et al. observed the highest prevalence of comorbidities in the BS-before-TKA population and did not take comorbidities into consideration, which may have confounded their results [29].

**Table 2:** Overview of complications analyzed in studies included in systematic review

Authors	Groups of patients analyzed as determined by study	Aggregate of complications observed for, reported, and/or analyzed			
Parvizi et al. [31]	14 patients who had BS before TJA	Hodgkin’s lymphoma, mortality (due to lung cancer), previous femoral fracture, superficial wound infection, patellofemoral pain, wound infection, bilateral deep vein thrombosis, revision needed, deep vein thrombosis, depression/para-suicide			
Kuklarni et al. [15]	53 patients who had BS prior TJA 90 patients who had BS after TJA	30-day complications Myocardial infarction Cerebrovascular accident *C. difficile* infection Renal failure Transient ischemic attack Lower respiratory tract infection Joint infection Dislocation Readmission Revision	90-day complications Mortality deep vein thrombosis Pulmonary embolus	18-month complications Mortality hip dislocation Dislocation Readmission Revision	1-year complication Hip revision
Severson et al. [37]	39 patients who had TKA before BS 25 patients who had BS within 2 years prior TKA 61 patients who had BS > 2 years prior TKA	Urinary tract infections, deep vein thrombosis, manipulation of joint post-operative arrhythmia, post-operative wound dehiscence (requiring wound revision), post-operative respiratory distress (requiring admission to ICU), deep infections of knee, delayed wound healing, intraoperative lateral femoral condyle fracture, post-operative acute renal failure, pulmonary embolism, acute cholecystitis, post-operative myocardial infarction, post-operative respiratory failure, revisions resulting from stiffness, osteolysis/polywear, osteolysis, acute periprosthetic fracture of the tibia, acute hematogenous infection requiring polyethylene liner exchange, deep infections			
Werner et al. [43]	66,523 non-obese patients who had TKA without BS 11,294 morbidly obese patients who had TKA without BS 219 morbidly obese patients who had TKA post-BS	Major complications: cerebrovascular accident, diagnosis of post-operative infection, deep vein thrombosis, post-operative irrigation and debridement, acute myocardial infarction, respiratory failure, pulmonary embolism			
		Minor complications: acute renal failure, pneumonia, post-operative blood transfusion, urinary tract infection, stiffness and/or manipulation under anesthesia, stiffness requiring lysis of adhesions			
Inacio et al. [17]	69 patients who had BS > 2 years prior TJA 102 patients who had BS within 2 years prior TJA 11,032 patients who had TJA without BS	30-day complications Deep surgical site infection Superficial surgical site infection Death Pulmonary embolism Deep vein thrombosis Any cause of revision Septic cause of revision Readmission	90-day complications Deep surgical site infection Superficial surgical site infection Death Pulmonary embolism Deep vein thrombosis Any cause of revision Septic cause of revision Readmission	1-year complications Deep surgical site infection Superficial surgical site infection Death Pulmonary embolism Deep vein thrombosis Any cause of revision Septic cause of revision	
Martin et al. [22]	91 patients who had BS before TKA 91 patients (with a BMI comparable to the BMI of the patients pre-BS) who had TKA with no BS 182 patients (with a BMI comparable to the BMI of the patients post-BS) who had TKA with no BS	Death, heart failure, myocardial infarction, deep vein thrombosis, pulmonary embolus, respiratory failure, pneumonia, urinary tract infection, acute renal failure, stroke, revisions			
Nickel et al. [29]	5914 patients who had BS before TKA 6480 patients with a BMI > 40 with no BS before TKA 26,616 patients with a BMI < 25 with no BS before TKA	30-day complications Death Stroke Pneumonia Myocardial infarction Deep vein thrombosis Pulmonary embolus Urinary tract infection Heart failure Acute renal failure Respiratory failure	90-day complications Periprosthetic infection Vascular/neuro injury Manipulation of joint Revision Extensor rupture	Minimum 2-year complications Periprosthetic infection Vascular/neuro injury Manipulation of joint Revision Extensor rupture	
Nearing et al. [28]	36 patients who had TJA before BS 66 patients who had TJA after BS	Surgical site infection, hematoma, venous thromboembolism, bleed requiring transfusion, periprosthetic infection, re-interventions (including revision, re-operation, manipulation, dislocation)			
McLawhorn et al. [24]	2636 patients who had BS prior TKA 2636 morbidly obese patients who did not have BS prior TKA	Stroke, mechanical complication of joint (including dislocation, loosening, breaking of implant, periprosthetic osteolysis), infection/inflammatory response of any kind (wound related or other post-operative infection/inflammatory response), sepsis, urinary tract infection, pneumonia, ileus, pulmonary embolism, deep vein thrombosis, revisions			
Schwarzkopf et al. [36]	1017 patients who had BS prior TKA	90-day complications Pulmonary embolism, deep vein thrombosis, acute myocardial infarction, respiratory failure, cerebrovascular event, urinary tract infection, blood transfusion, cardiac complications, peripheral vascular disease, respiratory complications, gastrointestinal complications, pneumonia, acute renal failure, acute cholecystitis, central nervous system problems, hematoma/seroma, wound dehiscence, post-operative infection, post-operative anemia			
Lee et al. [19]	70 patients who had BS prior TKA 86,539 patients who had only TKA	0.5-year revision risk Revision for PJI	1-year revision risk Revision for PJI	2-year revision risk Revision for PJI	5-year revision risk Revision for PJI
Liu et al. [20]	1478 patients who had BS prior TKA 60,259 patients who had obesity, no BS prior to TKA 281,973 patients who had no obesity or BS prior to TKA	30- and 90-day, 1-year readmission rate PJI, Dislocation, osteoarthritis, atrial fibrillation, cellulitis and abscess of leg, hematoma, septicemia, periprosthetic fracture			
Watts et al. [42]	47 patients who had BS prior to THA 94 patients who did not have BS prior to THA	Up to 5-year re-operation, revision surgery and PJI			

*BMI* body mass index, *BS* bariatric surgery, *PJI* periprosthetic joint infection, *THA* total hip arthroplasty, *TKA* total knee arthroplasty, *TJA* total joint arthroplasty

One study (7.6%) used descriptive statistics to evaluate whether the amount of time between BS and subsequent TJA affected 30-day complication rates [17]. Complication rates were 1.5% (95% CI: 0.0–4.3) for those who had BS more than 2 years prior to TJA, 2% (95% CI: 0.0–4.7) for those who had received BS within 2 years prior to TJA, and 2% (95% CI: 1.7–2.2) for those who had a BMI greater than 40 or a BMI greater than 35 with osteoarthritis and one additional comorbidity (Table 2) [17].

### Short-Term (90-Day) Complications

In total, five studies (38.4%) investigated 90-day short-term complication rates [15, 20, 24, 36, 43]. Two studies (15.4%) found lower 90-day complication rates for patients who underwent BS before TKA compared to a morbidly obese control population [24, 43]. McLawhorn et al. reported decreased odds (OR = 0.61, 95% CI: 0.45–0.84) of 90-day complications including infection, sepsis, deep vein thrombosis (DVT), and pulmonary embolism (PE) in patients who underwent BS before TKA, although there was no decreased odds of 90-day complications in THA patients (OR = 0.86, 95% CI: 0.50–1.48) [24]. Werner et al. categorized complications based on severity and reported significantly lower rates of major complications (9.6 vs. 19.0%, OR = 0.45, *p* = 0.001) and minor complications (15.1 vs. 22.6%, OR = 0.61, *p* = 0.01) for the patient groups who underwent BS before TKA [43]. Non-obese individuals had significantly lower rates of major complications (6.1%) and minor complications (8.3%) than both the morbidly obese and BS groups [43], suggesting that BS may not entirely mitigate the risks of previous morbid obesity. Further, BMI data at the time of TJA were not available, as both of these studies relied on administrative and billing databases. Three studies (23.1%) found no impact, positive or negative, of BS on 90-day TKA complications [15, 20, 36].

Three studies (25%) evaluated whether the amount of time between BS and TJA affects 90-day complication rates [17, 36, 37]. Severson et al. [37] reported no significant difference in 90-day complication rates between patients who had TKA before BS (21%), BS less than 2 years before TKA (4%), and BS more than 2 years before TKA (16%). Similarly, Schwarzkopf et al. determined no difference in 90-day TKA complications based on time from BS to TKA [36]. Inacio et al. [17] did not perform a statistical analysis that allowed comparison of 90-day complication rates across groups but reported complication rates for patients who underwent BS less than 2 years before TJA (2.0%), BS more than 2 years before TJA (1.5%), and morbidly obese controls (2.7%).

### Complications After at Least 1 Year of Follow-Up

Six studies (46.2%) documented the 1- to 2-year post-operative complication rates [17, 19, 20, 29, 31, 42]. One study reported rates of 1-year post-operative complications, defined as death, surgical site infection, DVT, PE, and/or revisions based on the timing of BS and TJA (Table 2) [17]. The incidence of post-operative complications was 2.9% (95% CI 0.0–6.9) in patients with BS greater than 2 years prior to TKA, 5.9% (95% CI: 1.3–10.4) for patients who received BS within 2 years prior to TKA, and 4.1% (95% CI: 3.8–4.5) for patients with morbid obesity. No statistical comparison between groups was performed.

Nickel et al. evaluated complications at a minimum 2-year follow-up [29]. Compared to morbidly obese patients, those who had BS before TKA had higher rates of periprosthetic joint infection (*p* = 0.002), manipulation (*p* < 0.001), extensor rupture (*p* < 0.001), and osteolysis (*p* = 0.049). Neurovascular injury occurred at similar rates between groups [29].

### Mortality Rate

Three studies (23.1%) reported mortality rates for TKA [29] or TKA and THA combined (Table 2) [24, 27]. Inacio et al. [17] observed no deaths within 90 days for those who underwent BS more than 2 years prior to TKA (*n* = 69) and those who underwent BS less than 2 years prior to TKA (*n* = 102) [17]. Kulkarni et al. reported one death within 90 days in 90 patients who underwent BS before TJA and no deaths in 53 patients who underwent BS after TJA [15]. Nickel et al. identified a higher mortality rate in patients who underwent BS before TKA (0.22%) compared with patients who underwent TKA without BS, both in the low BMI (0.08%, OR 2.85, *p* = 0.037) and high BMI (0.06%, OR 3.90, *p* < 0.001) groups [29].

### Revision Rate

Ten studies (76.9%) reported revision rates [15, 17, 19, 20, 22, 24, 28, 29, 31, 36]. Five studies [15, 20, 24, 28, 31] found no increase in re-interventions for patients who underwent BS before TKA. McLawhorn et al. included TKA performed in New York State between 1997 and 2011, and the authors did not identify a significant difference in revision rates between those receiving pre-operative BS and morbidly obese non-bariatric patients (hazard ratio [HR]: 0.90; 95% CI: 0.69–1.17; *p* = 0.431) [24]. When TKA revision was required, BS patients had a longer mean time between index TKA and revision TKA compared to obese patients who had not undergone BS (mean: 831 ± 759 vs 635 ± 656 days, *p* = 0.038). For THA patients in this study, there were no differences in revision, dislocation, or fracture rates [24]. At 1-year follow-up, Kulkarni et al. [15] found no TKAs required revision in patients who had BS prior to TKA (*n* = 37) or patients who had BS after TKA (*n* = 31) [15]. Nearing et al. found that timing of BS (before or after TJA) did not significantly affect revision and re-operation rates [28]. In populations of patients who underwent BS and THA or TKA, a small case series showed a 0% revision rate in 12 TKAs in 7 patients, with a mean follow-up of 3.7 years (range: 2–11 years) [31]. Comparing patients who underwent BS to morbidly obese patients prior to THA, Watts et al. saw a significant reduction in 1-, 2-, 5-, and 10-year revision risk [42].

Four studies (30.7%) [17, 19, 22, 29] suggested that patients who underwent BS before TKA were at higher risk for re-interventions. Martin et al. [22] found that the BS-before-TKA group had greater risk of re-operation compared to the high-BMI group (HR: 2.55, *p* = 0.02) and low-BMI group (HR: 2.4, *p* = 0.02). The BS-before-TKA group had greater risk of revision than the low-BMI group (HR: 2.2, *p* = 0.04), but not the high-BMI group (HR: 1.39, 95% CI: 0.4 to 4.7; *p* = 0.57). Nickel et al. [29] showed patients who underwent BS before TKA had a greater revision rate (7.38%) than both high-BMI (4.83%, OR: 1.57, *p* < 0.001) and low-BMI patients (2.52%, OR: 3.09, *p* < 0.001) who did not undergo BS [29]. While no statistical analysis was performed, Inacio et al. observed a trend toward higher revision rates in patients with BS greater than 2 years prior to TJA (3.4/100 years of observation), than in patients with BS within 2 years of TJA (2.7/100 years of observation), and those without BS (1.0/100 years of observation). It should be noted that these rates include THA [17].

### Hospital Length of Stay

Hospital LOS was reported in four studies (30.7%) [15, 17, 28, 37], of which one study [28] found statistically significant differences between groups. Nearing et al. [28] found that LOS was greater for patients who had TKA/THA before BS (3.8 ± 1.4 days) than after BS (2.9 ± 0.7 days; *p* = 0.0002). Severson et al. [37] found no significant difference in LOS days for patients who underwent TKA before BS (6.1 ± 2.3 days), patients who underwent TKA or 2 years or less after BS (5.7 ± 1.9 days), and patients who underwent TKA more than 2 years after BS (6.0 ± 3.0 days) [37]. Kulkarni et al. reported LOS of 7 days for the general TJA population, compared with 6.7 and 6.2 days for patients undergoing TJA before and after BS, respectively. No statistical comparison was performed, and lengths of stay for TKA and THA were not separated [15]. Finally, Inacio et al. showed LOS of 2.7 days (SD 0.8) for patients who had BS more than 2 years before TJA and 3 days (SD 1.4) for morbidly obese patients who did not undergo BS [17]. Again, THA was included and no statistical difference was found between groups.

## Discussion

Patients with a BMI of more than 40 face a relative risk ratio for TKA more than 30 times higher than individuals whose BMI is less than 25 [4]. Surgical weight loss is the most effective means by which to reduce excess weight in obese patients, and it has been shown to reduce obesity-related comorbid conditions and prolong life [1, 8]. As there are associations both between obesity and the risk for lower-extremity TJA and between obesity and complications after TJA, it is important to understand whether or not obesity is a modifiable risk factor prior to TJA. The purpose of this systematic review was to investigate the current literature to determine whether BS is associated with positive or negative effects on the outcomes of TKA and THA. We found that across a number of different outcomes, including revision, 30-day, 90-day, and other mid-term complications, there were no consistent results across studies. One study found reduced in-hospital complications for TKA and THA patients who had undergone prior BS [24].

There were several limitations in the studies included in this review. First, no prospective studies or randomized controlled trials (RCTs) have been published. Although RCTs would be ideal, they may not be pragmatic. Furthermore, insufficient power to detect differences between groups due to small sample sizes and infrequent event outcomes were major limitations of many studies. The inability to consider these studies as truly independent due to overlapping patient populations, as well as the heterogeneity in study design and methods for selecting/grouping patients, were other weaknesses. In addition, different types of BS and individual operative complications pose different risks of macro/micro-nutrient deficiencies and could affect the complication rate reported. Finally, the studies in this review should have addressed the question of the success rate of BS in the treatment of morbid obesity.

Regarding the risk of revision TJA, several studies documented no increased risk for re-operation in this patient population [15, 24, 28, 31]. Three studies found a higher revision rate in patients with BS and TKA when compared to a low-BMI group undergoing TKA [19, 22, 29]. Only one study showed reduced risk for THA revision in BS patients [42].

Studies examining the effect of BS on complication rates within 30 days of TJA likewise have produced mixed results [15, 19, 24, 28, 29]. In-hospital complications might occur less frequently in those who had BS before TKA and THA than in morbidly obese patients [24]. Three studies [15, 17, 28] comprising 233 patients found no difference in 30-day complications when patients were stratified by time interval between BS and TKA, including patients who had TKA before BS. The largest study evaluating the effect of BS on 30-day complications comprised 5914 patients and found that complications were most frequent in patients who had BS before TKA. Of note, a potential confounder is that preexisting comorbidities were also more frequent in this group.

The effect of BS on complication rates within 90 days of TJA also showed variation across the studies [15, 17, 19, 24, 29, 36, 37, 43]. Two studies [24, 43] found that patients who underwent BS before TKA had lower rates of complications, two studies showcased no benefit of BS prior to TKA for 90-day complications [15, 36], and one study [29] showed potential of BS resulting in an increase in complications. When looking at timing in THA/TKA, three studies showed no difference in 90-day complications based on BS [15, 36, 37]. McLawhorn et al. showed no advantage for prior BS with regard to 90-day complication risk for THA [24].

Overall, we found that there is no consensus based upon the current evidence for BS utility prior to lower-extremity TJA. However, each study should be critically analyzed. In several of the comparative studies, imbalance in concomitant comorbidities across study groups may have confounded their analyses and biased them against the BS cohorts, as it is well known that morbidly obese patients undergoing BS tend to have a higher baseline comorbidity burden than morbidly obese patients not undergoing BS [32, 33]. For example, Nickel et al. found that patients who underwent BS before TKA had a greater risk of complications than either non-obese or obese controls who did not undergo BS [29]. McLawhorn et al. used propensity score matching to balance the comorbid conditions in their study groups prior to surgical inventions, thereby reducing the risk for bias in their analysis [24]. Ultimately, prospective trials are necessary to corroborate or refute risks and benefits from these observational studies.

Studies reporting complications beyond 90 days were inadequate to inform surgical decision-making regarding TKA [17, 29, 31, 36]. Limitations include a lack of statistical testing [31] and lack of separation of TKA from THA [17]. Nickel et al. found that patients who had undergone BS had higher rates of infection, revision, manipulation, extensor rupture, and osteolysis [29]. However, these outcomes occurred infrequently overall and the odds of each complication were less than twice those of patients with BMI of over 40. Nickel et al. also found some increase in mortality rates among patients who underwent BS before TKA, though mortality rates were under 0.25% in all groups [29]. Some of this increased risk, both in rates of complications and death, is likely due to higher rates of comorbidities observed in the BS group. The absence of consistent findings regarding complication rates, small effect sizes where differences were identified, and absence of data on long-term functional outcomes makes these data less actionable.

LOS serves as a proxy for patients’ progress immediately after surgery, including any peri-operative complications. In our review, it appeared that BS had no significant effect on hospital LOS.

Given that obese patients make up an increasing proportion of patients receiving TKA, risk optimization in this more medically complex patient population is clinically important. Obese patients are more likely to experience complications including superficial and deep infection, acute kidney injury, cardiac arrest, and re-operation after TJA [12, 41]. Obesity also independently increases medical costs associated with TKA [14]. BS has been evaluated as a way to potentially reduce the risk of operating on these patients, but given the risks of a second elective surgery, it is imperative that orthopedic surgeons make recommendations to their patients based on an understanding of the current literature.

No studies have evaluated whether reduced biomechanical strain on the knee after BS eliminated the need for TJA in a subset of patients. Elevated mobility and exercise after BS may increase the number of patients requiring subsequent TKA [40]. Yet, this remains controversial; other studies suggest that weight loss after BS is associated with reduced knee complaints [10]. Further studies are required evaluating the proportion of people who require TJA following BS. It would also be beneficial to compare BS to non-surgical weight loss before TJA to determine how outcomes differ [16]. Lastly, there is growing favorability for performing sleeve gastrectomy over laparoscopic Roux-en-Y gastric bypass in the field of BS [25]. Yet, it is unknown how this switch in preference of BS type affects TJAs. Therefore, additional research is needed into the different types of BS and their resulting impact on TJAs.

The literature remains conflicted on the impact of BS on early, short- and long-term post-operative complications after TJA. Well-matched, observational studies may further our understanding of the impact of BS on TJA outcomes. In particular, the effect of the different BS types on TJA outcomes has yet to be elucidated. Ideally, prospective studies with higher levels of evidence are required to make more definitive conclusions as to the effects of BS on TJA.

## Electronic supplementary material

ESM 1 (DOC 34 kb)

ESM 2 (PDF 1.19 mb)

ESM 3 (PDF 1.19 mb)

ESM 4 (PDF 1.19 mb)

ESM 5 (PDF 1.19 mb)

ESM 6 (PDF 1.19 mb)

ESM 7 (PDF 1.19 mb)
