# Supplementary material for: The Effect of Bariatric Surgery Prior to Lower-Extremity Total Joint Arthroplasty: A Systematic Review
Source: HSS J. 2019 Apr 9;15(2):190–200. doi: 10.1007/s11420-019-09674-2 (PMC6609675; doi:10.1007/s11420-019-09674-2)
Supplement: Supplementary file 1 — (DOC 34 kb) [file 11420_2019_9674_MOESM1_ESM.doc]

**Appendix 1:** *Search Strategy*.

| **Database** | **PubMed/MEDLINE, Cochrane, Embase, ClinicalTrials.gov** |
| --- | --- |
| **Date** | September 2018 |
| **Strategy** | #1 AND #2 |
| **Limit** | Human AND English |
| **#1** | ((“Bariatric Surgery”[tw]) OR (“gastric bypass” [tw]) OR (“gastric band”) OR (“lap band” [tw]) OR (“gastric sleeve” [tw]) OR (“duodenal switch” [tw]) OR (“Roux-En-Y” [tw]) OR (“sleeve” [tw]) OR (“surgical weight loss” [tw]) OR (“Bariatric” [tw])) |
| **#2** | (("Arthroplasty, Replacement, Knee"[Mesh]) OR (total knee arthroplasty[tw]) OR (tka[tw]) OR (Total knee replacement[tw]) OR (TKR[tw]) OR ("Arthroplasty, Replacement, Hip"[Mesh]) OR (total hip arthroplasty[tw]) OR (tha[tw]) OR (Total hip replacement[tw]) OR (THR[tw])) |
